# Inosine in Neurodegenerative Diseases: From the Bench to the Bedside

**DOI:** 10.3390/molecules27144644

**Published:** 2022-07-21

**Authors:** Maria Sofia Basile, Placido Bramanti, Emanuela Mazzon

**Affiliations:** IRCCS Centro Neurolesi “Bonino-Pulejo”, Via Provinciale Palermo, Contrada Casazza, 98124 Messina, Italy; mariasofia.basile@irccsme.it (M.S.B.); placido.bramanti@irccsme.it (P.B.)

**Keywords:** inosine, neurodegenerative diseases, urate, Alzheimer′s disease, Parkinson’s disease, amyotrophic lateral sclerosis, multiple sclerosis

## Abstract

Neurodegenerative diseases, such as Alzheimer′s disease (AD), Parkinson’s disease (PD), amyotrophic lateral sclerosis (ALS), and multiple sclerosis (MS), currently represent major unmet medical needs. Therefore, novel therapeutic strategies are needed in order to improve patients’ quality of life and prognosis. Since oxidative stress can be strongly involved in neurodegenerative diseases, the potential use of inosine, known for its antioxidant properties, in this context deserves particular attention. The protective action of inosine treatment could be mediated by its metabolite urate. Here, we review the current preclinical and clinical studies investigating the use of inosine in AD, PD, ALS, and MS. The most important properties of inosine seem to be its antioxidant action and its ability to raise urate levels and to increase energetic resources by improving ATP availability. Inosine appears to be generally safe and well tolerated; however, the possible formation of kidney stones should be monitored, and data on its effectiveness should be further explored since, so far, they have been controversial. Overall, inosine could be a promising potential strategy in the management of neurodegenerative diseases, and additional studies are needed in order to further investigate its safety and efficacy and its use as a complementary therapy along with other approved drugs.

## 1. Introduction

It is believed that oxidative stress plays a key role in the initiation and progression of different neurodegenerative diseases, including Alzheimer′s disease (AD), Parkinson’s disease (PD), amyotrophic lateral sclerosis (ALS), and multiple sclerosis (MS) [1]. Oxidative stress occurs in the presence of an unbalanced redox state, which can be due to exaggerated amounts of oxidants, such as reactive oxygen species (ROS) and reactive nitrogen species (RNS), or to the impaired function of the antioxidant system [1]. The brain is particularly sensitive to oxidative stress [1]. Indeed, it consumes 20% of the body’s total oxygen supply, and a major part of that oxygen is converted to ROS [1]. Many ROS or RNS are also produced in the brain from specific neurochemical reactions, such as dopamine oxidation [1]. Moreover, the brain has many lipids, including myelin, which are susceptible to oxidative stress, and with aging, there is enhanced deposition of metal ions, which act as catalysts for ROS and RNS production [1].

Considering that oxidative stress is thought to be a promising target in neurodegenerative diseases and that antioxidant strategies could represent a valuable therapeutic approach, the potential use of inosine treatment in this context deserves particular attention.

**Figure 1:**
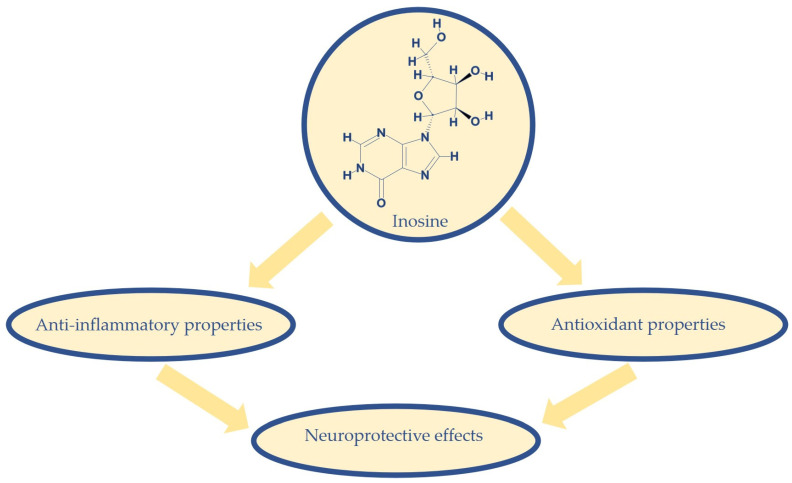
Inosine (molecular formula: C_10_H_12_N_4_O_5_) can exert neuroprotective effects that might be due to its anti-inflammatory and antioxidant properties.

Indeed, the purine nucleoside inosine (IUPAC name: 9-[(2*R*,3*R*,4*S*,5*R*)-3,4-dihydroxy-5-(hydroxymethyl)oxolan-2-yl]-1*H*-purin-6-one; molecular formula: C_10_H_12_N_4_O_5_), which has different intracellular roles and serves as an extracellular modulatory signal, can exert neuroprotective effects that might depend on its antioxidant and anti-inflammatory properties (Figure 1) [2,3]. Nowadays, there is growing evidence that inosine can regulate inflammatory processes and neuronal growth properties that might be significant for neurodegenerative disorders [4].

Interestingly, it has been suggested that inosine may potentially exert a therapeutic effect on disease processes implicated in neurological and psychiatric disorders [5].

Inosine is a precursor of urate, and it is consumed by many athletes as a dietary supplement to increase their performance, even though scientific studies do not support this belief [6,7]. 

Of note, inosine pranobex is a synthetic compound composed of inosine and the p-acetamido-benzoate salt of *N–N* dimethylamino-2-propanol, which has shown immunomodulatory and antiviral properties [8]. To date, this drug, which was first approved in 1971, is prescribed in many countries for the treatment of different viral diseases, such as herpes simplex virus and varicella infections, human papilloma virus, subacute sclerosing panencephalitis, cytomegalovirus, and Epstein–Barr virus infections, acute viral respiratory infections, measles, and immunosuppressed states [8].

Inosine is available as an oral supplement that can raise blood levels of urate and can also pass through the blood–brain barrier and increase cerebrospinal fluid (CSF) levels of urate [7,9]. While urate seems to be quickly degraded in the intestinal tract by bacterial flora, the oral administration of inosine can rapidly increase serum urate [10]. 

The protective action of inosine treatment could be mediated by its metabolite urate, which is the anionic form of uric acid (UA) and an end product of purine metabolism and has strong antioxidant properties, representing nearly 60% of the plasma antioxidant capacity [3,11].

Moreover, urate can improve neurotoxin-induced behavioral deficits and substantia nigra cell loss by interfering with GSK-3β activation and neurotoxin-induced Akt inhibition [11].

In addition, urate can induce autophagy, which is involved in the clearance of intraneuronal aggregated α-synuclein, by inhibiting the mammalian target of rapamycin (mTOR) [11]. 

Urate can be found both intracellularly and in bodily fluids as the anionic form of UA (2,6,8-trioxy-purine), and its blood concentration depends not only on urate biosynthesis (e.g., oxidation through the enzyme xanthine oxidoreductase) and on urate excretion but also on dietary intake [12]. Hypotheses about the role of urate seem to be controversial. On the one hand, urate can be a pathogenic factor in gout, urolithiasis, and nephropathy, and hyperuricemia has been linked to cardiovascular diseases and other disorders [13,14]. However, the eventual risk of urate nephropathy can be reduced by high fluid consumption and a potassium-rich alkaline diet [14]. Moreover, people with a homozygous deficiency for genetic xanthine oxidoreductase (XOR), who have low-to-no UA serum levels, show a total absence of central nervous symptoms [15]. On the other hand, it is known that urate can exert strong antioxidant properties and may have a potential neuroprotective effect, promoting neuronal function and integrity [13]. Several lines of evidence have shown that patients with neurodegenerative disorders tend to have low serum, CSF, or brain tissue UA levels [15]. Interestingly, urate levels have been associated with a decreased risk of developing PD and AD [16]. Additionally, urate levels have been linked to favorable progression in PD, ALS, and multiple system atrophy [16]. Overall, the potential protective action of urate has assumed particular relevance for central nervous system (CNS) homeostasis and diseases [13]. Of note, several studies have shown the neuroprotective effects of urate in neurological disorders, and several lines of evidence support the idea that urate-mediated mechanisms may be a bridge between different neurodegenerative processes [13,16]. 

Interestingly, it has been shown that inosine administration can augment serum urate levels with reasonable safety in humans [17]. Furthermore, it has been found that inosine activates adenosine receptors (A1, A2A, A2B, and A3), resulting in different effects based on the biological context [5] Indeed, while the A_1_ and A_3_ receptors mainly interact with members of the Gi/o family of G proteins, decreasing the intracellular levels of cyclic adenosine monophosphate (cAMP), the A2A and A2B receptors interact with members of the Gs family of G proteins, increasing intracellular cAMP [5]. 

Moreover, inosine can increase energetic resources by improving the availability of ATP [18]. In particular, inosine can be shuttled to the pentose phosphate pathway via ribose-1-phosphate and, after that, to glycolysis, generating NADH, ATP, and subsequently, lactate [19].

Overall, these data lay the groundwork for investigating the use of inosine in neurodegenerative diseases. Several preclinical studies and clinical trials investigating the effects of the administration of inosine in the treatment of neurodegenerative diseases have been performed or are underway.

**Figure 2:**
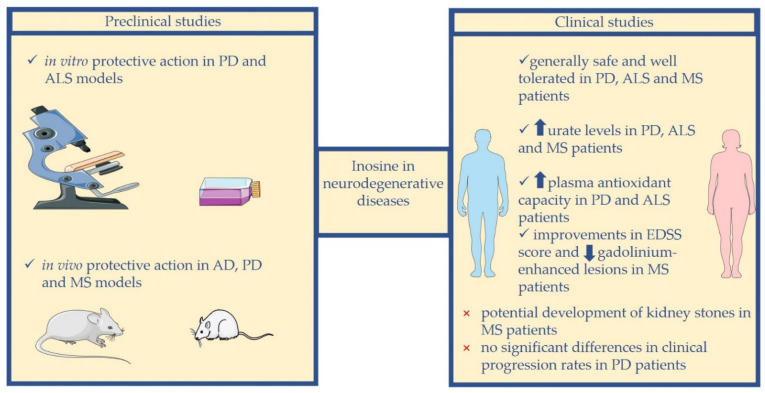
Overview of the most important results from preclinical and clinical studies investigating the potential use of inosine in neurodegenerative diseases.

In this review, we discuss the possible use of inosine as a preventive or therapeutic strategy in the management of neurodegenerative diseases, and we report the current preclinical and clinical studies on this topic, focusing our attention on AD, PD, ALS, and MS (Figure 2).

## 2. Inosine in AD

AD is a neurodegenerative disorder that affects more than 45 million people worldwide and represents the most frequent form of dementia [20].

AD is more frequent in people above the age of 65 years, and the possibility of developing AD rises considerably with age and doubles every 5 years thereafter [21]. Age is the most important risk factor for AD, thus suggesting that since life expectancy is rising over time, the number of AD patients will also gradually increase [21].

Even though AD is primarily sporadic, mutations in three genes, named amyloid precursor protein (APP), presenilin 1 (PSEN1), and presenilin 2 (PSEN2), can cause a rare familial form of AD, which develops earlier compared to sporadic AD, commonly between 30 and 50 years of age [20].

AD is characterized by β-amyloid (Aβ)-containing extracellular plaques and tau-containing intracellular neurofibrillary tangles [22].

Although AD usually manifests with prominent amnestic cognitive impairment, it can less frequently present as non-amnestic cognitive impairment [22]. Short-term memory difficulty represents the most frequent AD presentation; however, defects in expressive speech, visuospatial processing, and executive functions can also appear [22].

The diagnosis of AD relies on clinical presentation fulfilling different criteria and on fluid and imaging biomarkers [23]. To date, there is no efficacious treatment able to reverse or slow AD progression [21]. Currently, there are six drugs approved by the US Food and Drug Administration (FDA): aducanumab, donepezil, galantamine, rivastigmine, memantine, and a manufactured combination of memantine and donepezil [21]. Except for aducanumab, which targets Aβ plaques, the others act as symptomatologic treatment at two levels: via the agonism of the cholinergic system or the antagonism of the N-methyl-D-aspartate receptor (NMDA receptor) [21].

Different factors have been suggested to be involved in AD pathogenesis, including oxidative–nitrative stress, endoplasmic reticulum stress, mitochondrial dysfunction, inflammatory cytokines, pro-apoptotic proteins, and altered neurotransmitter levels [24].

In particular, it has been shown that oxidative stress might play a key role in the pathogenesis of AD [25]. Indeed, it is associated with amyloid pathology and tau pathology, creating a vicious pathophysiological cycle promoting mitochondrial dysfunction and metal toxicity [25]. Hence, oxidative stress can be considered a promising biomarker and therapeutic target for AD, and the potential use of antioxidants should be considered as a potential useful strategy for AD treatment [25].

Interestingly, the levels of the well-known antioxidant urate were associated with a decreased risk of developing AD [16]. In particular, it was shown that gout was associated with a reduced risk of AD [16]. Moreover, it was shown that urate levels in cortical and striatal tissues had a decreasing trend in AD in comparison to controls in males [26].

In addition, Jiang et al. showed the lack of inosine in serum of senescence-accelerated mouse prone 8 (SAMP8) mice, a murine model of age-related learning and memory deficits and AD, thus suggesting that the anti-inflammatory, immunomodulatory, and neuroprotective functions of inosine could be reduced in SAMP8 [27]. Therefore, the authors suggested the hypothesis that the lack of inosine could contribute to a rise in pro-inflammatory cytokines and to the immunodeficiency of SAMP8 and could lead to learning and memory deficits in SAMP8 [27]. On the other hand, González-Domínguez et al. found augmented levels of inosine in serum from APP/PS1 mice, a transgenic model of AD [28].

Of note, Nielsen et al. found that inosine serum levels were reduced in AD patients [29]. Moreover, Alonso-Andrés et al. demonstrated that the frontal cortex from the early stages of AD show apparently reduced levels of inosine, whereas the parietal cortex and temporal cortex show significantly higher levels of inosine, at least at certain stages of AD [30].

Interestingly, Waugh suggested that the supplemental use of ascorbic acid associated with metabolic precursors to uric acid, such as inosine, could prevent or reduce the progression of AD and amnestic mild cognitive impairment [31].

So far, there have been no clinical studies investigating the possibility of using inosine treatment in AD patients. Thus, here, we describe the data from preclinical studies on this topic.

### Preclinical Studies

Teixeira et al. analyzed the effects of inosine treatment at different doses on behavioral and neurochemical parameters in a streptozotocin (STZ)-induced rat model of AD [32]. In this study, rats were divided into four groups (control; STZ; STZ plus inosine at a dose of 50 mg/kg; and STZ plus inosine at a dose of 100 mg/kg) [32]. Rats received STZ by bilateral intracerebroventricular injection, and they were treated intraperitoneally with inosine for a period of 25 days [32]. Of note, inosine treatment, either at a dose of 50 mg/kg or at a dose of 100 mg/kg, was able to prevent memory impairment caused by STZ [32].

Moreover, it was shown that inosine could improve memory dysfunctions by interacting with different targets in brain regions implicated in cognitive functions, including cholinergic enzymes, ion pump activities, and redox status [32]. Indeed, STZ induced a rise in Na^+^, K^+^-ATPase and Mg-ATPase activities and a reduction in the Ca^2+^-ATPase activity in the hippocampus and cerebral cortex, and it has been shown that inosine might prevent these changes in ion pump activities [32]. Moreover, inosine was able to prevent the rise in acetylcholinesterase (AChE) activity and the changes in AChE and choline acetyltransferase (ChAT) expression caused by STZ [32]. In addition, inosine exerted a protective effect against oxidative alterations due to STZ in the brain [32].

Furthermore, recently, Teixeira et al. explored inosine action in the STZ-induced rat model of AD, focusing on the analysis of its effects on memory, neurotrophic factors, neuroinflammatory cytokines, purinergic receptor expression, and morphological changes in the rat hippocampus and cerebral cortex [33].

In particular, rats were distributed in four groups (control; STZ; STZ plus inosine at a dose of 50 mg/kg; and STZ plus inosine at a dose of 100 mg/kg) and were treated with intracerebroventricular injections of STZ or buffer [33]. Three days after the surgical procedure, they were treated with inosine (at a dose of 50 mg/kg or 100 mg/kg) for a period of 25 days [33].

Interestingly, inosine treatment prevented memory deficits, reduced the immunoreactivity of the brain A2A adenosine receptor induced by STZ, and enhanced the brain levels of anti-inflammatory cytokines as well as the expression of brain-derived neurotrophic factor and its receptor [33]. In addition, inosine was able to reduce the alterations induced by STZ in the molecular layer of the hippocampus and to protect against the decrease in immunoreactivity for synaptophysin induced by STZ in the CA3 hippocampal region [33]. On the other hand, inosine treatment was not able to prevent an increase in GFAP in rats treated with STZ [33]. Overall, these data suggest that inosine could be a promising therapeutic strategy for AD due to its ability to modulate different brain mechanisms implicated in neuroprotection [33].

In addition, Teixeira et al. also explored inosine action in a rat model of scopolamine-induced cognitive impairment [34]. In this model, scopolamine caused impairment in the memory acquisition and consolidation phases, and inosine prevented only the damage in memory consolidation [34]. Moreover, scopolamine enhanced the activity of acetylcholinesterase and decreased the activity of Na^+^, K^+^-ATPase, and inosine exerted a protective effect against these changes in the consolidation protocol [34].

Moreover, inosine ameliorated the redox status by decreasing the levels of reactive oxygen species and thiobarbituric acid-reactive substances and re-establishing antioxidant enzyme activity [34].

Overall, these data suggest that inosine can protect against scopolamine-induced memory consolidation impairment by regulating the brain redox status, cholinergic signaling, and ion pump activity and that it can be a promising strategy for the prevention of the neurodegenerative mechanisms implicated in AD [34].

## 3. Inosine in PD

PD is the most frequent movement disorder and the second most common neurodegenerative disorder of the CNS, affecting 1% of the population above 60 years and 1–2 per 1000 of the population at any time [35].

Less than 10% of PD cases are familial, whereas the majority of them are sporadic, and their appearance can be influenced by different variables, such as age, gender, and genetic and environmental factors [36]. Nearly 30% of familial PD cases and 3–5% of sporadic ones are monogenic forms, derived from a single mutation in a gene that can be inherited dominantly (e.g., *SNCA* and *LRRK2)* or recessively (e.g., *Parkin*, *PINK1*, *DJ-1*, and *ATP13A2)* [37]. Of note, all familial PD cases seem to be associated with mutations in genes that can be directly or indirectly implicated in mitochondrial dysfunction [36].

PD is characterized by different motor and non-motor symptoms [38]. The cardinal motor symptoms are tremor, rigidity, bradykinesia/akinesia, and postural instability [38]. Moreover, PD is characterized by the loss of dopaminergic neurons in the substantia nigra pars compacta and by intracellular inclusions, known as Lewy bodies, containing aggregates of misfolded α-synuclein [38].

Even though the diagnosis of PD is mainly clinical, specific investigations can facilitate the differential diagnosis from other forms of parkinsonism [38].

The gold-standard therapy in PD is represented by L-3,5-dihydroxyphenylalanine (L-DOPA) along with peripheral inhibitors of L-aromatic amino acid decarboxylase (LAAD) [39]. Although L-DOPA treatment has a good motor response in PD patients, in the long term, it becomes suboptimal due to the appearance of motor fluctuations and dyskinesias, and it does not stop or reduce PD progression, possibly even becoming harmful to the substantia nigra pars compacta dopaminergic neurons [39]. The long-term L-DOPA syndrome encouraged the design of other antiparkinsonian drugs, which could aid in postponing the beginning of L-DOPA treatment or be administered along with L-DOPA to improve motor response and/or diminish L-DOPA-induced dyskinesias [39]. Among these classes of drugs are: dopamine receptor agonists, anticholinergic drugs, catechol-oxy-methyltransferase (COMT) inhibitors, the N-methyl-D-aspartate (NMDA) receptor antagonist, amantadine, and type-B monoamine oxidase (MAO_B_) inhibitors (e.g., selegiline, rasagiline, and safinamide) [39].

Numerous preclinical and clinical studies have investigated the possible use of inosine in PD treatment. These studies stemmed from converging biological, epidemiological, and clinical data that suggested urate increase as a potential strategy to delay PD progression [10]. Indeed, numerous PD biochemical features (e.g., mitochondrial dysfunction, reduced nigral glutathione levels, and increased nigral iron load) can be correlated with higher oxidative stress, thus suggesting that urate could exert a neuroprotective effect in PD by reducing oxidative nigral injury [40]. Of note, increasing or decreasing the levels of urate respectively protects or worsens PD phenotypes in rodent models of PD [40]. Zhang et al. demonstrated that urate could exert protective effects on dopaminergic cells against oxidative stress by affecting Nrf2 signaling [41]. Gong et al. showed that urate can exert a neuroprotective effect on dopaminergic neurons in a rat model of PD, possibly mediated by the regulation of Akt/GSK3β signaling [42]. Nakashima and colleagues found that UA has a neuroprotective effect on dopaminergic neuronal loss, ameliorating motor dysfunction and PD development in unilateral 6-hydroxydopamine-lesioned mice fed on a diet with 1% UA and 2.5% a uricase inhibitor (potassium oxonate) in order to cause hyperuricemia [43].

Moreover, it has been found that higher blood levels of urate were associated with a reduced risk of PD, and increased serum and CSF urate baseline levels can be correlated with slower rates of clinical decline [44,45,46,47]. Interestingly, it was shown that there was a trend toward decreased urate levels in PD male patients’ cortical and striatal tissues compared to controls and a significant increase in urate levels in male control tissues [26]. Furthermore, men on a urate-increasing diet or with gout have a decreased probability of developing PD [1]. In addition, high consumption of dairy products may be associated with a higher risk of PD in men, possibly linked to the uricosuric effect exerted by milk protein [17].

Here, we present findings from preclinical and clinical studies that have explored the possibility of using inosine in PD.

### 3.1. Preclinical Studies

Cipriani et al. evaluated whether inosine could exert protective effects in rodent MES 23.5 dopaminergic cells against oxidative stress in an in vitro model of PD and to what extent its effects could be due to urate [3]. The authors analyzed inosine in MES 23.5 cells cultured either alone or along with cortical astrocytes (MES 23.5–astrocyte co-cultures) and treated with 200 µM H_2_O_2_ [3]. They found that when dopaminergic cells were cultured with astrocytes, the H_2_O_2_ toxic effect was decreased compared with when they were cultured alone [3]. Furthermore, in MES 23.5–astrocyte co-cultures, free radical generation and oxidative damage were diminished [3]. Experiments with conditioned medium showed that the protective action of inosine could be associated with the release of a protective factor from inosine-stimulated astrocytes [3]. However, urate levels were not significantly elevated following inosine treatment, whereas other purine metabolites, including adenosine, hypoxanthine, and xanthine, significantly increased [3]. Overall, these results suggest that inosine may exert a protective effect in PD independently of urate-mediated effects [3].

El-Shamarka et al. investigated the potential disease-modifying action of inosine in a rotenone-induced PD mouse model [2]. Mice were divided into six groups: a normal control group that received dimethylsulfoxide, a PD control group that received rotenone, a group that was treated with L-dopa/carbidopa along with rotenone, and three treatment groups that were treated with low, medium, and high doses of inosine along with rotenone [2]. The behavioral, biochemical, and histologic findings from this study revealed that inosine administration exerted a dose-dependent protective effect against PD progression, with effects comparable to standard treatment with L-dopa/carbidopa [2]. This antiparkinsonian action of inosine might be due to the inhibition of oxido-nitrosative stress, of neuroinflammation, and of ERK phosphorylation and to the down-regulation of A2AR expression [2].

### 3.2. Clinical Studies

The Safety of Urate Elevation in PD (SURE-PD) study (NCT00833690) is a phase II randomized, double-blind, placebo-controlled, dose-ranging trial aimed at evaluating the safety, tolerability, and urate-elevating capability of oral inosine treatment and defining the optimal dosing schedule and its suitability for phase III evaluation in PD. The study involved 75 participants with early PD not yet requiring symptomatic treatment and with a serum urate concentration lower than 6 mg/dL (approximately the population median value) for up to 2 years. Participants were randomly divided into three groups: placebo or inosine, dosed to result in a mild (6.1–7.0 mg/dL) or moderate (7.1–8.0 mg/dL) serum urate increase. One to six capsules of 500 mg of placebo or inosine were administered orally per day (in up to three divided doses). The rate of serious adverse events was the same or lower in the inosine groups compared to placebo [10]. Nobody developed gout, and three participants of the inosine group had symptomatic urolithiasis [10]. Serum urate increased by 2.3 and 3.0 mg/dL in the two inosine groups compared to placebo, and the urate level in the CSF was higher in both inosine groups [10]. Moreover, secondary analyses showed the nonfutility of inosine for delaying disability [10]. These results demonstrated that inosine was clinically safe, tolerable, and effective in increasing serum and CSF urate levels in early PD patients, thus suggesting a potential disease-modifying benefit of inosine in PD [10]. Moreover, it was found that oral inosine treatment in early PD patients from the SURE-PD trial increased serum urate without elevating blood pressure [48].

Another study evaluated whether oral inosine may determine changes in the plasma and CSF antioxidant capacity by measuring ferric-reducing antioxidant power (FRAP), and the authors also evaluated urinary markers of oxidative injury, in particular, 8-hydroxydeoxyguanosine (8-OHdG), in patients with early PD who participated in the SURE-PD trial [40]. Long-term oral inosine administration in early PD patients caused a dose-dependent, persistent increase in plasma antioxidant capacity associated with its urate-increasing effects, whereas CSF antioxidant capacity and urine 8-OHdG did not show differences [40]. Moreover, the plasma FRAP increase was found to be inversely correlated with the rate of clinical decline [40].

Furthermore, a post hoc analysis of sex differences in SURE-PD was conducted in order to discover whether women and men with PD showed differences in their biochemical and clinical responses to inosine treatment [49]. Interestingly, this study showed Class II evidence that inosine led to higher serum and CSF urate increases in women than in men with early PD and might delay PD progression, slowing the rate of Unified PD Rating Scale (UPDRS) change, more in women than in men [49]. The difference in the inosine-induced serum urate increase may be associated with the sex difference in baseline serum urate levels, which is approximately 1 mg/dL lower in women, potentially consequent to estrogen effects on the renal tubule [49].

In another study, Iwaki et al. investigated the safety and efficacy of inosine in PD patients in an Asian population [50]. They found that inosine effectively increased serum urate levels in patients at lower doses than those reported in other European and American studies, and they did not record any severe side effects [50].

The clinical trial Study of Urate Elevation in PD, Phase 3 (SURE-PD3, NCT02642393) was a multicenter, randomized, double-blind, placebo-controlled, phase 3 trial that involved 298 early PD patients with serum urate below the population median value (<5.8 mg/dL). Although 92% of the participants completed the study, the study was closed prematurely due to a prespecified interim futility analysis [51]. No significant differences in clinical progression rates or in secondary efficacy outcomes between participants in the inosine group and those in the placebo group were observed [51]. Moreover, participants randomized to inosine showed a sustained increase in serum urate of 2.03 mg/dL and fewer severe adverse events but more kidney stones than participants randomized to placebo [51]. Therefore, among recently diagnosed PD patients, inosine treatment, compared to placebo, did not significantly change the rate of clinical disease progression, thus not supporting the use of inosine as a treatment for early PD [51].

Moreover, Watanabe and colleagues conducted an open-label, single-arm, multicenter trial to investigate the efficacy and safety of the co-administration of febuxostat, a xanthine oxidoreductase inhibitor, and inosine in PD patients [15]. Patients received febuxostat 20 mg and inosine 500 mg twice a day for 8 weeks, and the primary endpoint was the change in the MDS-UPDRS Part III score immediately prior to and after 57 days of treatment [15]. After treatment, serum hypoxanthine levels were significantly increased, and the MDS-UPDRS Part III score was significantly decreased [15]. Overall, these data indicate that the co-treatment of febuxostat and inosine could be safe and effective in improving symptoms in PD patients [15].

## 4. Inosine in ALS

ALS is the most frequent motor neuron disease in adults, with an incidence of nearly 1–2.6 cases per 100 000 persons per year, an average age of onset of 58–60 years, and an average survival of 3–4 years from the onset of the disease to death [52]. Of all cases, 90–95% are sporadic, whereas 5–10% are hereditary and named familial ALS [52].

Approximately 60–80% of familial ALS patients have a mutation of large effect, such as *C9orf72* (40%), *SOD1* (20%), *FUS* (1–5%), and *TARBDP* (1–5%), which are the most frequent [53]. The genetics of sporadic ALS is less clear [53]. Results from twin studies have identified that the genetic contribution to sporadic ALS is 61%, and almost 10% of sporadic ALS patients show mutations in genes associated with familial ALS [53]. Multiple pathogenetic pathways, which involve oxidative stress, neuronal inflammation with immune cells infiltrating the CNS, mitochondrial dysfunction, RNA splicing errors, and errors in protein conformation, have been implicated in ALS [36].

ALS is a progressive neurodegenerative disease characterized by the degeneration of upper and lower motor neurons, resulting in motor and extra-motor symptoms [54]. The early onset of ALS can differ between patients [54]. Indeed, while some patients have a spinal-onset disease, which is characterized by muscle weakness of the limbs, others have a bulbar-onset disease, which is characterized by dysarthria and dysphagia [54]. Even though the primary symptoms of ALS are correlated with motor dysfunction (e.g., muscle weakness, spasticity, and dysphagia), many patients show cognitive and/or behavioral symptoms [54].

The diagnosis is mainly made by clinical examination along with nerve conduction studies, electromyography, and laboratory testing [55]. Nowadays, the mainstay of care for ALS patients is prompt intervention to manage symptoms, such as using nasogastric feeding, preventing aspiration, and providing ventilatory support [56].

There are no efficacious therapies for ALS, and only two drugs, which provide modest benefits in mortality and/or function, are approved by the FDA for the treatment of ALS: riluzole and edaravone [36,57]. Riluzole is a glutamate antagonist that acts by decreasing excitotoxicity, whereas edaravone is an antioxidant and free radical scavenger that can decrease oxidative stress and cell death [36,57].

Although the biological mechanisms that lead to ALS are not fully clarified, both autopsy and laboratory studies have shown that they can include damage to motor neurons from oxidative stress, thus laying the groundwork for the use of edaravone in ALS and for the possibility that antioxidants in general, including urate or agents able to raise urate levels (e.g., inosine), might be neuroprotective in ALS [9,58]. Of note, blood urate levels are lower in ALS patients than in healthy controls [16]. Several studies, but not all, have shown that high urate levels are associated with better survival in ALS [9]. In addition to the neuroprotective action mediated by a urate increase, inosine could boost reinnervation in ALS [9]. Furthermore, different studies have revealed that metabolic dysfunction is a pivotal mechanism involved in the pathogenesis of ALS and that it might influence the rate of disease progression [19]. Enhanced inosine metabolism could be protective in ALS since inosine supplementation might raise ALS fibroblast mitochondrial spare respiratory capacity and glycolytic flux and capacity [19].

Here, we report the results from preclinical and clinical studies that have analyzed the possibility of using inosine in ALS.

### 4.1. Preclinical Studies

Dysfunctional energy metabolism has been shown to have an unfavorable effect on disease progression in ALS [59]. Allen and colleagues analyzed the basis of the catabolic defect in ALS through an innovative phenotypic metabolic array by profiling fibroblasts and induced neuronal progenitor-derived human induced astrocytes from *C9orf72* ALS patients in comparison to controls and evaluating the rates of production of reduced nicotinamide adenine dinucleotides from 91 energy substrates [59]. They found that *C9orf72* induced astrocytes and fibroblasts showed an adenosine-to-inosine deamination defect due to a reduction in adenosine deaminase, which is also present in induced astrocytes from sporadic patients [59]. Interestingly, the use of inosine supplementation to overcome the adenosine deaminase defect exerted a positive effect in vitro, enhancing glycolytic energy output and improving motor neuron survival in co-cultures with induced astrocytes [59]. Overall, this study shows that inosine supplementation, together with the regulation of the level of adenosine deaminase, may be a beneficial therapeutic strategy in ALS patients [59].

Another study further supported the hypothesis that increased inosine metabolism could be protective in ALS since inosine supplementation enhanced ALS fibroblast mitochondrial spare respiratory capacity and glycolytic flux and capacity [19]. Indeed, by analyzing the metabolic profile of fibroblasts derived from ALS patients in comparison to controls, it was shown that there was a positive correlation between inosine metabolism and disease progression in familial ALS cases [19]. Furthermore, inosine supplementation in ALS fibroblasts was found to be bioenergetically favorable [19].

### 4.2. Clinical Studies

The clinical trial NCT02288091 is a single-center, open-label study aimed at evaluating the safety, tolerability, and urate-elevating capability of inosine in ALS patients and determining whether inosine treatment can modulate the levels of oxidative stress and damage biomarkers [60]. In particular, these included the levels of 3-nitrotyrosine (3-NT), glutathione, and FRAP, as these values have been shown to be modified in ALS [60]. 3-NT is a marker of tyrosine nitration mediated by RNS, whose levels were previously found to be increased in SOD1 transgenic mice and ALS patients [60].

This study involved 25 ALS patients with lower serum urate levels than the population median value of 5.5 mg and lasted 12 weeks [60]. A total of 96% of the participants completed the study [60]. The dose of inosine was titrated to reach serum urate levels of 7–8 mg/dL [60]. These target levels of serum urate were obtained in 6 weeks [60]. No significant adverse events, including those of special concern (urolithiasis and gout), related to inosine were recorded [60]. Moreover, the investigated biomarkers of oxidative stress and damage underwent significant changes that suggested significant biological effects of inosine [60]. Indeed, inosine treatment led to higher antioxidant capacity and lower 3-NT levels in plasma [60]. The higher plasma antioxidant capacity may be associated with the increase in serum urate levels, consistent with the fact that urate is an important contributor to FRAP [60].

Overall, it has been shown that inosine appears to be safe, well tolerated, and capable of increasing serum urate levels in ALS patients [60]. Therefore, these data contribute to providing the rationale for other clinical trials aimed at evaluating inosine as a potential therapeutic strategy for ALS patients [60].

## 5. Inosine in MS

MS is the most frequent chronic neurodegenerative, inflammatory, and demyelinating disease of the CNS in young adults, with an age of onset between 20 years and 40 years and a global prevalence of 35.9 per 100,000 people in 2020 [61,62].

Although the causes underlying MS are not clear, it is known that MS is associated with different genetic factors, such as major histocompatibility complex *HLA-DRB1* locus, and environmental factors, including tobacco smoking, vitamin D levels, UV radiation, Epstein–Barr infection [63].

MS is a multifactorial immune-mediated disorder that is characterized by the accumulation of demyelinating lesions that appear in the white and gray matter of the brain and spinal cord and shows heterogeneous clinical manifestations and courses [61].

There are four clinical courses of MS: relapsing–remitting MS (RRMS), which is characterized by the appearance of relapses at irregular intervals with complete or incomplete neurological recovery and is present in nearly 85% of patients; secondary progressive MS (SPMS), which occurs in approximately 2–3% of RRMS patients each year and is characterized by progressive, irreversible disability, independent of the presence of relapses; primary progressive MS (PPMS), which occurs in ~10–15% of patients and is characterized by disease progression from the onset that results in gradual, progressive, and permanent neurological deficits for more than 1 year without relapses; and progressive relapsing MS (PRMS), which is uncommon and is characterized by progressive disease, with acute relapses and periods of continuous progression between relapses [61]. Moreover, clinically isolated syndrome (CIS) has been introduced to indicate patients who first show features of inflammatory demyelination that might be MS without fulfilling its diagnostic criteria [61]. Furthermore, each MS subtype can be further defined as active or not active depending on the appearance of relapses or lesions identified by magnetic resonance imaging (MRI) [61].

The diagnosis of MS relies on a combination of clinical, radiographic, and laboratory data and is based on the demonstration of the dissemination of MS disease features in space and time [63].

Currently, MS is treated with a multidisciplinary approach that involves disease-modifying therapies (DMTs), symptomatic treatment, lifestyle changes, psychological assistance, and rehabilitative strategies [63]. As of July 2020, nine classes of DMTs were approved for MS treatment: interferons, glatiramer acetate, teriflunomide, sphingosine 1-phosphate [S1P] receptor modulators, fumarates, cladribine, natalizumab, ocrelizumab, and alemtuzumab [63].

Oxidative stress is considered to be involved in the pathogenesis of MS [64]. It is known that active MS plaques usually include inflammatory cells expressing intracellular inducible nitric oxide synthase (iNOS) and generating peroxynitrite-dependent radicals [6]. Hence, inosine has been considered a natural antioxidant that could be useful in the treatment of MS, at least by increasing the tissue levels of UA as a peroxynitrite scavenger [2]. Several studies in animal models have investigated the role of peroxynitrite-dependent radicals in the development of CNS lesions and have shown the therapeutic action of the peroxynitrite-dependent radical scavenger UA [6].

Furthermore, it has been demonstrated that MS patients often show lower serum UA levels than controls and that there is an inverse correlation between the occurrence of MS and serum UA levels [6]. However, an increase in circulating concentrations of inosine and UA in MS patients has also been shown [65]. In addition, it has been demonstrated that MS patients with relapse showed significantly decreased serum UA levels compared to MS patients with remission and that MS patients with blood–brain barrier (BBB) disruption showed significantly decreased serum UA levels compared to MS patients with normal BBB [66]. Statistical evidence has shown that gout with hyperuricemia and MS are almost mutually exclusive conditions, thus suggesting that hyperuricemia could be protective against MS [67]. Interestingly, it has been shown that in pairs of both mono- and dizygotic twins with only one sibling affected by MS, blood UA levels were lower in the twins with MS compared to their healthy twins [68]. Furthermore, it has been shown that during therapy with drugs known to be effective in MS, such as glatiramer acetate, interferon β (IFNβ), high-dose methylprednisolone, and natalizumab, there is an increase in serum UA levels [69].

Considering these data, increasing serum UA levels in MS patients by treatment with a UA precursor such as inosine could be a possible therapeutic strategy in MS [4].

Here, we describe the data of preclinical and clinical studies that have investigated the possibility of using inosine in MS.

### 5.1. Preclinical Studies

Scott et al. evaluated the effects of inosine and inosinic acid on the pathogenesis of an in vivo model of MS, experimental allergic encephalomyelitis (EAE) [4]. The administration of inosine or inosinic acid greatly increased serum UA levels, while only a minor, transient rise in serum inosine was found, thus suggesting that inosine is quickly metabolized to UA [4]. Moreover, inosinic acid treatment suppressed the occurrence of clinical signs of EAE and favored recovery from the disease [4]. In particular, the beneficial effect on EAE mice was observed to be associated with higher UA levels, but not with inosine levels, in CNS tissue [4]. Hence, the authors suggested that the inosine and inosinic acid mechanism of action in EAE is mediated by their metabolism to UA [4].

Junqueira and colleagues also investigated the action of inosine on the development and progression of EAE [70]. Inosine was administered intraperitoneally at a dose of 1 or 10 mg/kg twice a day for 40 days in EAE mice [70]. The authors found that inosine treatment had neuroprotective effects in EAE mice, regulating the onset of the disease and preventing its progression by decreasing the severity of clinical symptoms, including thermal and mechanical hyperalgesia and weight loss, which are characteristic of the disease [70]. The beneficial action of inosine was linked to its anti-inflammatory effects via the inhibition of inflammatory cell entry into the CNS and a decrease in IL-17 levels in peripheral lymphoid tissue, along with the prevention of demyelination lesions and the inhibition of astrogliosis in the spinal cord [70]. Moreover, inosine was able to prevent A2AR up-regulation in the spinal cord, probably via an ERK1-independent pathway [70]. Overall, these data revealed the effectiveness of inosine in the blockade of EAE development and progression and in the inhibition of neuroinflammation and demyelinating processes, thus suggesting a potential neuroprotective effect of inosine in MS [70].

### 5.2. Clinical Studies

Spitsin et al. conducted a pilot study aimed at increasing the serum levels of UA in patients with MS [71]. The oral administration of UA did not raise low serum levels of UA, owing to its degradation by gastrointestinal bacteria, whereas the oral administration of inosine increased serum UA levels and maintained these raised levels for a year or more without side effects [71]. Moreover, 3 out of 11 patients receiving inosine presented some evidence of clinical improvement, and no signs of disease progression appeared in the other patients [71].

Another clinical study was conducted in order to assess the safety and efficacy of oral inosine as a single drug treatment in MS patients [72]. This study showed that 32 MS patients treated with oral administration of 1–2 g of inosine daily (given twice) for a period of nearly 3 years had lower relapse rates compared to 32 non-treated MS patients used as controls, without adverse effects [72]. Moreover, the non-treated MS patients had a higher increase in the Expanded Disability Status Scale (EDSS) score than the treated patients [72]. As regards the serum UA levels, it was observed that after the follow-up period, treated MS patients without relapses presented significantly increased serum UA levels compared to treated MS patients with one or more relapses and non-treated MS patients [72]. Moreover, treated MS patients with relapses showed significantly increased serum UA levels compared to non-treated patients [72]. Overall, these data suggest that therapeutic strategies aimed at increasing serum UA levels could be beneficial for some MS patients [72].

Furthermore, a clinical study (NCT00067327) was carried out with the primary aim of assessing the safety and tolerability of inosine in RRMS patients and with the secondary objectives of evaluating the effects of inosine treatment on serum urate levels, the progression of neurologic disability, the number of new active lesions on MRI, and the variations in serum levels of inflammatory markers [6]. Sixteen RRMS patients were selected for this randomized and double-blinded trial and were treated with the oral administration of inosine for a period of one year [6]. The trial included two arms: a placebo-controlled crossover study consisting of 6 months of placebo followed by 6 months of inosine, and inosine treatment for 12 months after baseline assessment [6]. To reach a target serum urate level of 6–9 mg/dL, the inosine dosage was first 1–2 g/day and increased by 0.5 g/day at biweekly intervals, and for the majority of patients, the inosine dosage necessary to maintain target urate levels was between 2 and 3 g/day [6]. Higher serum urate levels were associated with a significant reduction in the number of gadolinium-enhanced lesions and with improvements in the EDSS score [6]. Moreover, inosine treatment changed several MRI intensity-based parameters, in some cases correlating with serum UA level alterations [6]. The results of this study suggest that using oral administration of inosine to increase serum urate levels could be beneficial for at least some MS patients [6]. Notably, the only side effect correlated with inosine treatment was the formation of kidney stones, which occurred in 4/16 subjects in this study [6].

The ASsociation of Inosine and IFNβ in RRMS (ASIIMS) trial was a concept, multicenter, double-blind, placebo-controlled trial aimed at investigating whether combined therapy with IFNβ and inosine could have more efficacy than IFNβ alone on the accumulation of disability in patients with RRMS [69]. RRMS patients on IFNβ for at least 6 months were randomly divided into two treatment groups: IFNβ + inosine (experimental) and IFNβ + placebo (control) for a period of 2 years [69]. The inosine dosage was adapted to maintain serum UA levels in the range of asymptomatic hyperuricemia (≤10 mg/dL) [69]. The combination of IFNβ and inosine was found to be safe and well tolerated; however, this combined therapy did not show any additional advantage for preventing the accumulation of disability in comparison with IFNβ alone [69]. In light of these results, the authors suggested that, although this combined treatment failed to show further benefits, a possible neuroprotective role of UA in MS should not necessarily be excluded, but it should be considered that the endogenous neuroprotective mechanisms involved in MS are complex and that UA may not be the only actor in this scenario [69].

In this line of research, a randomized, double-blind, placebo-controlled trial was conducted with the primary endpoints of evaluating the safety and tolerability of inosine in combination with interferon B-1a (IFNB-1a) in RRMS patients and with the secondary endpoint of assessing the effectiveness of this combined treatment by analyzing clinical and radiological parameters [18]. RRMS patients starting treatment with IFNB-1a at a dose of 44 µg three times per week were randomly assigned to be treated with either inosine 3 g/day or placebo in a double-blind manner for a follow-up period of 12 months [18]. Among the 36 enrolled patients, 2 patients belonging to the inosine group had UA serum levels higher than 10 mg/mL and symptoms caused by renal colic, not leading to hospital admission, whereas 10 patients showed asymptomatic hyperuricemia (>7 mg) [18]. The clinical and radiological parameters analyzed to evaluate treatment efficacy were similar between the groups, and there were no patients who progressed to SPMS [18]. Overall, inosine treatment was associated with hyperuricemia and renal colic, with no effects on MS pathology [18]. However, a trend of fatigue reduction was found in patients treated with inosine, suggesting that additional studies should investigate this potential effect, which could be related to a possible increase in energetic resources and improvement in ATP availability [18].

## 6. Discussion

Neurodegenerative diseases, such as AD, PD, ALS, and MS, currently represent critical unmet medical needs. Thus, discovering novel therapeutic strategies in this field should be a priority of utmost importance. Considering that oxidative stress could play a key role in the initiation and progression of different neurodegenerative diseases, the potential use of inosine, known for its antioxidant properties, deserves particular attention. The protective action of inosine treatment could be mediated by its metabolite urate. Although higher urate levels can cause gout and urolithiasis and may be associated with cardiovascular and other disorders, urate can exert strong antioxidant effects and may have potential neuroprotective action, promoting neuronal function and integrity, thus suggesting that urate-mediated mechanisms could be a bridge between different neurodegenerative processes [13,16]. Moreover, it has been shown that patients with neurodegenerative disorders tend to have low serum, CSF, or brain tissue UA levels and that urate levels can be linked to favorable progression in certain neurodegenerative diseases [15,16]. In addition, it has been shown that inosine administration can increase serum urate levels with reasonable safety in humans [17].

On the basis of these data, inosine seems to be a valuable potential therapeutic strategy for neurodegenerative diseases. A noteworthy advantage is that inosine is a low-cost supplement. Indeed, the cost for a monthly supply of inosine is nearly USD 10–60, depending on the brand and the dose of inosine necessary to achieve the required blood levels of urate [9]. Furthermore, due to its ready availability as an over-the-counter supplement, inosine might represent a quite simple strategy for symptomatic relief and possible treatment of different neurological impairments [73]. In this review, we discuss current preclinical and clinical studies investigating the use of inosine in AD, PD, ALS, and MS, and we provide a schematic overview of the most important preclinical and clinical evidence in this field in Table 1.

As regards AD, to date, there are no clinical studies analyzing the effects of inosine treatment in AD patients. However, preclinical evidence suggests that inosine might be a promising therapeutic strategy for AD thanks to its ability to modulate different brain mechanisms involved in neuroprotection [33].

Indeed, it was shown that inosine improves memory dysfunctions in an experimental rat model of AD, possibly by interacting with different molecular targets in brain regions implicated in cognitive functions (e.g., cholinergic enzymes, ion pump activities, and redox status) [32]. Moreover, the use of inosine in an experimental rat model of AD was also found to be able to prevent memory deficits, reduce the immunoreactivity of the brain A2A adenosine receptor, and increase the brain levels of anti-inflammatory cytokines as well as the expression of brain-derived neurotrophic factor and its receptor [33]. In addition, inosine was able to reduce alterations in the molecular layer of the hippocampus and to protect against the decrease in immunoreactivity for synaptophysin in the CA3 hippocampal region [33].

In addition, it has also been shown that inosine can exert a protective effect against scopolamine-induced memory consolidation impairment through the regulation of brain redox status, cholinergic signaling, and ion pump activity, thus suggesting that it could be useful in the prevention of the neurodegenerative mechanisms implicated in AD [34].

The results from preclinical studies in PD suggest that inosine can exert a protective effect independently of urate and raise the possibility that the beneficial antiparkinsonian action of inosine might be due to its capability to reduce neuroinflammation and oxido-nitrosative stress, suppress ERK phosphorylation, and down-regulate A2AR expression [2,3]. Clinical studies exploring the use of inosine treatment in early PD patients have shown that inosine is generally safe, tolerable, and effective in increasing serum and CSF urate levels and can increase plasma antioxidant capacity [10,40]. However, no significant differences in clinical progression rates were found [51]. Moreover, it has been found that the co-treatment of febuxostat and inosine in PD patients significantly increased serum hypoxanthine levels, significantly decreased the MDS-UPDRS Part III score, and overall, could be safe and effective in improving symptoms in PD patients [15].

As regards ALS, preclinical studies have shown that inosine supplementation exerted a positive effect in vitro, increasing glycolytic energy output and improving motor neuron survival in co-cultures with induced astrocytes, and have also found that inosine supplementation in ALS fibroblasts was bioenergetically favorable and that inosine metabolism was positively correlated with disease progression in familial ALS cases [19,59]. Moreover, inosine appeared to be safe, well tolerated, and capable of raising serum urate levels, increasing plasma antioxidant capacity, and decreasing 3-NT plasma levels in ALS patients [60].

Concerning MS, in vivo studies have demonstrated that inosine or inosinic acid can increase serum UA levels and can exert beneficial effects in EAE mice [4,70]. Moreover, it has been shown that inosine can increase serum UA levels and can reduce the relapse rate, the progression of disability, and the number of gadolinium-enhanced lesions in MS patients [6,71,72]. Although inosine treatment appeared to be generally well tolerated, the possible formation of kidney stones could be a side effect [6]. Moreover, one study showed that combined therapy with IFNβ and inosine in RRMS patients was safe and well tolerated but did not show any additional advantages for the accumulation of disability in comparison with IFNβ alone [69]. On the other hand, another study demonstrated that inosine in combination with IFNB-1a in RRMS patients was associated with an increase in adverse events (e.g., renal colic) and was not effective despite the trend of fatigue reduction found in patients in the inosine group [18].

According to the results of current preclinical and clinical studies exploring the possible use of inosine in neurodegenerative diseases, the most important properties of inosine seem to be its antioxidant action and its capability to raise urate levels and to increase energetic resources by improving ATP availability. Inosine seems to be generally safe and well tolerated; however, the possible formation of kidney stones should be carefully monitored, and data concerning its effectiveness should be further investigated since, so far, they have been controversial.

To date, in this line of research, different studies evaluating the effects of inosine in the treatment of other neurodegenerative diseases, such as Huntington’s disease (HD), multiple system atrophy, and superficial siderosis, have also been conducted.

In particular, it has been shown that inosine reduced 3-nitropropionic acid (3-NP)-induced HD-like symptoms in rats, at least in part, by activating the A2AR/BDNF/TrKB/ERK/CREB signaling pathway [74].

Furthermore, the clinical trial NCT03403309 showed that inosine 5’-monophosphate treatment was generally safe and well-tolerated in patients with multiple system atrophy [75]. Additionally, the clinical trial NCT04890808, which is not yet recruiting, aims to evaluate whether oral inosine or inositol hexaphosphate could be possible effective long-term therapies to stop or delay neural damage progression in superficial siderosis.

## 7. Conclusions

Overall, inosine could be a promising potential preventive or therapeutic strategy in the management of neurodegenerative diseases thanks to its antioxidant action and its ability to raise urate levels and to increase energetic resources by improving ATP availability. However, additional studies are strongly needed in order to further explore its safety and efficacy and its possible use as a complementary therapy along with other approved drugs in neurodegenerative diseases.

## Figures and Tables

**Table 1:** Overview of the most important preclinical and clinical evidence on the use of inosine in AD, PD, ALS, and MS.

Neurodegenerative Diseases	Preclinical Evidence	Clinical Evidence
Alzheimer′s disease	Inosine improved memory dysfunctions in a rat model of AD by interacting with different targets in brain regions implicated in cognitive functions (e.g., cholinergic enzymes, ion pump activities, and redox status) [32].	Not present.
	Inosine treatment in a rat model of AD was able to: prevent memory deficits; reduce the immunoreactivity of the brain A2A adenosine receptor; increase the brain levels of anti-inflammatory cytokines and the expression of brain-derived neurotrophic factor and its receptor; reduce alterations in the molecular layer of the hippocampus; and protect against the decrease in immunoreactivity for synaptophysin [33].	
	Inosine protected against scopolamine-induced memory consolidation impairment through the regulation of brain redox status, cholinergic signaling, and ion pump activity [34].	
Parkinson’s disease	Inosine exerted a protective action independent of urate in a cellular model of PD [3].	Inosine was clinically safe, tolerable, and effective in increasing serum and CSF urate levels in early PD patients [10].
		Inosine administration in early PD patients caused a dose-dependent, persistent increase in plasma antioxidant capacity [40].
	Inosine exerted a dose-dependent protective effect in a mouse model of PD*,* possibly due to the inhibition of oxido-nitrosative stress, of neuroinflammation, and of ERK phosphorylation and to the down-regulation of A2AR expression [2].	Among recently diagnosed PD patients, inosine treatment did not significantly change the rate of clinical disease progression [51].
		Co-treatment of febuxostat and inosine was relatively safe and effective in PD patients [15]. After treatment, serum hypoxanthine levels were significantly increased, and the MDS-UPDRS Part III score was significantly decreased [15].
Amyotrophic lateral sclerosis	Inosine exerted a positive effect in vitro, increasing glycolytic energy output and improving motor neuron survival in co-cultures with induced astrocytes [59].	Inosine appeared to be safe, well tolerated, and capable of increasing serum urate levels in ALS patients and led to higher antioxidant capacity and lower 3-NT levels in plasma [60].
	Inosine supplementation in ALS fibroblasts was bioenergetically favorable [19].	
Multiple sclerosis	Administration of inosine or inosinic acid in EAE mice increased serum UA levels [4]. Inosinic acid treatment suppressed the occurrence of clinical signs of EAE and favored recovery from disease [4].	Inosine increased serum UA levels and maintained these increased levels for a year or more without side effects in MS patients [71]. Three of eleven patients receiving inosine presented some evidence of clinical improvement, and no signs of disease progression appeared in the others [71].
		MS patients treated with inosine had lower relapse rates without adverse effects [72].
		Inosine treatment in RRMS patients increased urate levels [6]. Higher serum urate levels were associated with a significant reduction in the number of gadolinium-enhanced lesions and with improvements in the EDSS score [6]. The only side effect was the formation of kidney stones in 4/16 subjects [6].
	Inosine had neuroprotective effects in EAE mice, blocking EAE development and progression and inhibiting neuroinflammation and demyelinating processes [70].	Combined therapy with IFNβ and inosine in RRMS patients was safe and well tolerated but did not show any additional advantage for the accumulation of disability in comparison with IFNβ alone [69].
		Inosine administration together with IFNB-1a was associated with hyperuricemia and renal colic and was not effective in RRMS patients, but it was associated with a trend of fatigue reduction [18].
